# Some New(?) Uses for Carbolic Acid

**Published:** 1887-01

**Authors:** 


### EDITORIAL DEPARTMENT.

F. E. DANIEL, M. D., EDITOR AND PUBLISHER.

COLLABORATORS:

*E. J. Doering, M. D., Chicago.*
*Geo. Cupples, M. D., San Antonio.*
*Odo Betz, M. D., Germany.*
*E. J. Beall, M. D., Fort Worth, Texas.*
*T. C. Osborn, M. D., Cleburne, Texas.*
*Wm. Penny, M. D., New York.*
*H. O. Marcy, M. D., Boston.*
*C. K. Gregg, M. D., Mexico.*
*R. M. Swearingen, M. D., Austin.*
*C. H. Wilkinson, M. D., Galveston.*
*J. W. McLaughlin, M. D., Austin.*
*R. H. L. Bibb, M. D., Mexico.*

### SOME NEW (?) USES FOR CARBOLIC ACID.

The man who first made carbolic acid was, indeed, a benefactor to his race. Its uses are innumerable; and its value as a remedy is but half known and appreciated. There is no one remedy in the whole Pharmacopœa that has a wider range of application, nor which is adapted to so many, and such diverse pathological conditions. Its value is appreciated in many forms of disease—but we believe it is not generally known that in superficial inflammations it is almost a specific. We see Tannin—a saturated solution, much praised as an application to in-growing nails—but its effect is nothing to compare with the prompt and satisfactory action of pure carbolic acid. The inflamed part may be as tender and as sensitive as an inflamed eye, and one application of carbolic acid, poured or dropped from the vial directly on it, so as to penetrate under the nail, will relieve the pain almost instantly; and if applied at bedtime, next morning the part will be as insensible to the touch as wood. After this, the corner of the nail can be clipped off, or allowed to remain, and an occasional application will *prevent* the recurrence of the trouble.

Almost the same may be said with regard to bone-felons and ordinary boils. If applied early, carbolic acid not only will relieve the pain, but will prevent the formation of pus—will dissipate it. The same with those troublesome things called "sty"; but caution has to be observed here. The application is best made by a small pointed stick—a match-end, trimmed to a point. Carbolic acid is recommended for freckles, but the limited trials we have made of it in this trouble has not convinced us of any great value. The trouble is, it will destroy the epidermis, and with it, the pigment will be removed; but it is apt to leave a spot darker than the freckle.

Perhaps the greatest value carbolic acid possesses, in addition to its well known effect in hemorrhoids, is the power to remove *warts*—those ugly, horny excrescences which often disfigure ones hands. We first used the carbolic acid on an ugly old cracked-open, fissured wart, on a little girl's finger. Three applications caused the wart to drop off, leaving a clean, healthy surface, and there has been no return. Since then, we have removed many; some requiring only one application; and one of those ugly pedunculated warts on the face. We have had several to drop off on being touched once only; ugly, disfiguring moles also, can be gotten rid of by this means, though it occasionally requires several applications; and if the mole be situated on a tender part of the body, say the neck, it sometimes makes a sore for a day or so. We do not claim originality in the above; but we assert that we have never seen any mention of the use of carbolic acid in this connection, except for condylomata, and it was suggested to our mind by the sensation and effect produced by getting a drop or so on our hand, accidentally. To relieve itching, it is also powerful; but in most cases, the remedy is worse than the disease; not that it is painful, but for days, there is a sense of *drawing*, of tightness of the part which is uncomfortable.

A good deal has been said lately about the erythematous rash produced by Quinine. We are in our own person, a striking illustration of the truth of the claim that quinine will produce such a rash, The singular part of our case is, that the smallest quantity of quinine (or of bark, or of the tincture) will, in a few hours, cause a bright red eruption on the skin, in irregular circular patches, and they invariably make their appearance in the same places, and no where else, and of the same size and shape. These are attended with the most intense itching, such that *scratching* will allay only for a brief period. One of these torments comes in the palm of the hand on taking the minutest quantity of quinine. It was to this palmer rash that we first applied the carbolic acid, in a frantic endeavor to get rid of the intense pruritus. It was a success, but we were ten days in getting rid of the drawing sensation, and before desquamation took place.

To sprains and bruises, especially about joints, it is an admirable application, relieving pain at once, and preventing inflammation, and unless the skin be very tender one need not hesitate to paint the part *very lightly*, with the pure acid; it will rarely blister about the hands or feet.

Our readers will do well to try this remedy in in-growing nails. for we can assure them that it is a God-send in that painful affection, and as a wart-exterminator, it has no equal. We have not tried the much lauded "small doses of epsom salts," for we have no faith in any thing so contrary to reason and common sense.

